# Development and validation of the Crohn’s disease patient-reported outcomes signs and symptoms (CD-PRO/SS) diary

**DOI:** 10.1186/s41687-018-0044-7

**Published:** 2018-05-09

**Authors:** Peter D. R. Higgins, Gale Harding, Nancy K. Leidy, Kendra DeBusk, Donald L. Patrick, Hema N. Viswanathan, Kristina Fitzgerald, Sarah M. Donelson, Marcoli Cyrille, Brian G. Ortmeier, Hilary Wilson, Dennis A. Revicki, Gary Globe

**Affiliations:** 1 University of Michigan, Ann Arbor, MI USA; 2 Evidera, Bethesda, MD USA; 3 Genentech Inc., South San Francisco, CA USA; 4 University of Washington, Seattle, WA USA; 5 Amgen Inc., Thousand Oaks, CA USA; 6 IBD Program University of Michigan, SPC 5682, 1650 West Medical Center Drive, Ann Arbor, MI 48109 USA; 7 Present address: Allergan Inc., Irvine, CA USA

**Keywords:** Crohn’s disease, Patient-reported outcome, Signs and symptoms, Reliability, Validity, Clinical trial endpoints

## Abstract

**Background:**

The clinical course of Crohn’s disease (CD) and the effect of its treatment are monitored through patient-reported signs and symptoms (S&S), and endoscopic evidence of inflammation. The Crohn’s Disease Patient-reported Outcomes Signs and Symptoms (CD-PRO/SS) measure was developed to standardize the quantification of gastrointestinal S&S of CD through direct report from patient ratings.

**Methods:**

The CD-PRO/SS was developed based on data from concept elicitation (focus groups, interviews; *n* = 29), then refined through cognitive interviews of CD patients (*n* = 20). Measurement properties, including item-level statistics, scaling structure, reliability, and validity, were examined using secondary analyses of baseline and two-week clinical trial data of adults with moderate-to-severe CD (*n* = 238).

**Results:**

Findings from qualitative interviews identified nine S&S items covering bowel and abdominal symptoms. The final CD-PRO/SS daily diary includes two scales: Bowel S&S (three items) and Abdominal Symptoms (three items), each scored separately. Each scale showed evidence of adequate reliability (α = 0.74 and 0.67, respectively); reproducibility (intraclass correlation coefficient > 0.80), and validity, with the last including moderate correlations with the Inflammatory Bowel Disease Questionnaire bowel symptom score and select items (ranging from *r* = 0.43–0.54). Scores distinguished patients categorized by patient global ratings of disease severity (*p* < 0.0001).

**Conclusions:**

Results suggest the CD-PRO/SS is a reliable and valid measure of gastrointestinal symptom severity in CD patients. Additional longitudinal data are needed to evaluate the ability of the CD-PRO/SS scores to detect responsiveness and inform the selection of responder definitions.

**Electronic supplementary material:**

The online version of this article (10.1186/s41687-018-0044-7) contains supplementary material, which is available to authorized users.

## Significance of this study

### What is already known about this subject?

- The US Food and Drug Administration (FDA) has established a pathway for rigorous development of disease-specific Patient-reported Outcome (PRO) tools for clinical trials and clinical use.
- Currently, there are no measures developed and validated according to the FDA PRO guidance available to assess the symptoms of Crohn’s disease (CD).

### What are the new findings?

- Using the US FDA pathway for rigorous development of disease-specific PRO tools, we have developed and validated a new patient-reported sign and symptoms measure for clinical trials and clinical use in CD.
- This is the first symptom measure of CD to meet US FDA PRO guidelines.
- This modular instrument can be used with appropriate individual modules customized to the mechanism of action of a candidate therapy, from purely anti-inflammatory medications, to those targeting pain, dysmotility, or functional symptoms.

### How might it impact on clinical practice in the foreseeable future?

- Using electronic device systems, PROs in CD can be routinely measured before and between appointments in order to identify response to therapies or failure of therapies.

## Background

Crohn’s disease (CD) is a type of inflammatory bowel disease (IBD) that causes chronic inflammation of the gastrointestinal (GI) tract. While the incidence and prevalence of CD is subject to considerable variation both between and within geographic regions, the highest occurrence is generally in the developed countries of North America and Europe [1]. A recent study estimates that approximately 565,000 people in the United States (US) have CD [2], with the prevalence in Europe varying from 1.5 to 213 cases per 100,000 persons [1]. The incidence of CD is increasing, with a global annual incidence ranging from 3 to 20 per 100,000 person-years, depending on geographic region [3]; currently the highest incidence of CD in Europe is 12.7 per 100,000 person-years and 20.2 per 100,000 person-years in North America [4].

CD most commonly affects the most distal part of the small intestine (ileum) and large intestine (colon), but can develop in any part of the GI tract from the mouth to the rectum. Patients who have active CD often experience abdominal pain, fatigue, and diarrhea. While the cause of CD is unknown, risk factors include family history of the disease; it is most likely to present initially between ages 15–30 with a second peak of onset at ages 50–70 [5].

Clinically, CD is monitored through signs and symptoms of disease activity and periodic objective assessment, including endoscopy, imaging, or measurement of biomarkers to evaluate mucosal inflammation. In clinical trial settings, the Crohn’s Disease Activity Index (CDAI) [6] score has been used to assess disease activity, combining patient-reported signs and symptoms (loose/liquid stools, abdominal pain, general well-being) with clinical assessments (i.e., complications, presence of abdominal mass, change in weight, hematocrit levels, use of antidiarrheal agents), using a weighted scoring algorithm.

More recently, there has been growing interest for an approach that discriminates between different aspects of a disease, such as clinically derived signs, symptoms, and/or clinical tests. The US Food and Drug Administration (FDA) released a guidance for the development of Patient-Reported Outcome (PRO) measures to support labeling claims for new medical treatments and products [7]. This document emphasizes the importance of conducting qualitative research among the target population throughout the process of instrument development; this is to ensure that the measure is consistent with the patient experience and covers what patients consider most important about the condition and/or treatment intervention. Quantitative work to assess the instrument’s psychometric properties, such as reliability and validity, is also recommended. This standard in instrument development is increasingly seen as a regulatory requirement for efficacy evaluation and labeling purposes for treatment interventions [7, 8]. For these reasons, a new patient-reported sign and symptom measure for CD was developed and validated according to the US FDA PRO Guidance: this is the first symptom measure of CD to meet these guidelines.

The Crohn’s Disease Patient-reported Outcomes (CD-PRO) instrument was designed to comprehensively assess the signs, symptoms, and impact of CD through six modules. Modules 1 (Bowel Signs and Symptoms) and 2 (Abdominal Symptoms) comprise the CD-PRO Signs and Symptoms (CD-PRO/SS) measure. Module 3 addresses Systemic Symptoms, Module 4 addresses Coping Strategies, Module 5 addresses Daily Life Impact, and Module 6 covers Emotional Impact. Any or all of these modules may be used in any given study.

The focus of this paper is the CD-PRO/SS measure’s ability to evaluate treatment-related outcomes and support labeling claims related to the GI signs and symptoms of CD in clinical trials of adults (age 18 or older) with moderate-to-severe CD treated in outpatient settings. This paper describes the development and initial validation of this instrument. Given the day-to-day symptom variability characteristic of CD, the CD-PRO/SS is completed as a daily diary and is designed for electronic administration.

As noted throughout the paper, details related to the CD-PRO/SS development and validation are provided in the Additional file 1. Also included in this supplement is information on the Systemic Symptoms scale (Module 3 of the CD-PRO), a five-item scale that can be included as part of the daily diary to evaluate the non-gastrointestinal systemic symptoms of CD. Based on the qualitative work, these symptoms were found to be prevalent, relevant, and important to the patient. However, systemic symptoms are generally not affected by current gut-specific agents. From a regulatory perspective, such symptoms are considered “distal” to the target disease activity and are therefore less suitable for testing treatment effects and/or inclusion in a product label. Because the intent is to develop the CD-PRO/SS for use in drug development trials as a Drug Development Tool [9], Module 3 (Systemic Symptoms) is not included in the CD-PRO/SS measure. At the discretion of the user/sponsor, Module 3 can be administered as part of the diary and serve as an exploratory assessment in clinical trials. This scale may also be useful in studies or clinical trials evaluating the systemic component of CD. Information in the Additional file 1 is intended to facilitate use of this Module.

## Materials and methods

The research was conducted in two phases, consistent with the methodology outlined in the US FDA PRO Guidance [7]. Phase I addressed the content and structure of the measure and the documentation of content validity through qualitative research methods. Phase II addressed the quantitative properties of the measure, including scoring and tests of reliability and validity, based on a secondary analysis of baseline through Week 2 Phase II clinical trial data. All data collection and recruitment procedures met institutional review board (IRB) and Health Insurance Portability and Accountability Act requirements and all applicable state and federal laws and regulations, with study protocols approved by an independent IRB and written informed consent obtained from study subjects prior to completing any study related activities.

### Phase I: Qualitative – Development and content validity

A two-staged qualitative research process was used to determine instrument content and structure, and to ensure clarity and understanding in the target patient population. Focus groups and interviews were conducted using a semi-structured discussion guide, informed by clinical expert input and a review of the literature to cross reference symptoms, and were audio-recorded and transcribed for analysis. Additionally, participants completed a sociodemographic questionnaire for use in characterizing the study sample. For both stages of the qualitative research process, subjects were recruited from US gastroenterology clinics and included ambulatory adult patients with clinician-confirmed CD based on available biopsy. Patients participating in an interventional study were excluded, as were those with an ileostomy, colostomy, or an intra-abdominal surgery. Efforts were made to recruit patients who represented a range of disease activity, from mild to severe, based on the Sandler estimated CD Activity Index [10].

Additional methods are outlined below, with details provided in the online Additional file 1.

#### Stage 1: Focus groups and one-to-one interviews

Five focus groups (*n* = 20) and nine one-to-one qualitative interviews were conducted to identify important CD symptoms, explore the frequency and variability of these symptoms, and inform the development of response options and appropriate recall for a symptom measure in this target population. Subjects were recruited from seven US clinical sites to represent a range of races, ethnicities, geographic locations, and disease activity. Discussion focused on participants’ current symptom experiences, their experiences during an episode or flare-up, and the impact of these symptoms on their daily life.

Content analyses were performed by independent coders, with data organized using qualitative software (i.e., NVivo or ATLAS.ti). Participant quotes were grouped and summarized by thematic code to assess saturation of concepts. Saturation is defined as the point at which no substantially new themes, descriptions of a concept, or terms are introduced as additional discussions are conducted [11].

Results were discussed with clinical experts and used to generate a list of relevant symptoms, and a draft CD-PRO/SS questionnaire, including instructions, items, and response options.

#### Stage 2: Cognitive interviews

Two rounds of cognitive interviews (*n* = 20) were conducted to examine the relevance, comprehensiveness, and clarity of the draft CD-PRO/SS (including systemic symptoms), and to refine the measure as needed. Subjects were asked to complete the questionnaire independently and were then interviewed about the content, including instructions, recall period, candidate items, and response options. Upon completion of 16 interviews (Round 1), the instrument was edited for clarity based on subject comments, and the revised instrument was evaluated by a new sample of CD patients (Round 2, *n* = 4). Round 2 also provided an opportunity to examine patient understanding of the scales administered using an electronic handheld device. Upon completion of this set of interviews, the instrument was assessed for translatability and finalized for quantitative testing.

### Phase II: Quantitative – Score reliability and validity

A secondary analysis of data from a 24-week Phase II clinical trial evaluating the efficacy of an experimental, active treatment relative to placebo for the treatment of moderate-to-severe CD was conducted to further examine the properties of the CD-PRO/SS. Baseline and Week 2 data were used to determine the structure of the measure and scoring, and to examine reliability and validity. A total of 238 subjects were randomized to treatment and included in this analysis. Adult patients, ages 18–65, with a physician diagnosis of ileal, ileo-colonic, or colonic CD a minimum of six months prior to the baseline, with moderate-to-severe CD activity (CDAI score ≥ 220 and ≤ 450) and inadequate response to, loss of response to, or intolerance to either immunomodulators or anti-tumor necrosis factor (TNF) agents were eligible to participate in the study. Evidence of active inflammation was required, as demonstrated by at least one of the following: elevated C-reactive protein at screening (≥ 5 mg/L); elevated fecal calprotectin at screening (≥ 200 μg/g); or endoscopic evidence of inflammation within 12 weeks prior to baseline. Patients with clinical manifestations of short bowel syndrome, stricture with obstructive symptoms within three months prior to baseline, or evidence of non-inflammatory obstruction during the six months prior to baseline were excluded from the study.

#### Measures

Subjects completed the CD-PRO/SS (9 candidate items) and Module 3 Systemic Symptoms (5 candidate items) throughout the study using an electronic hand-held device given to the subject upon enrollment, with training provided by clinic site personnel. The daily diary scores from Baseline through Week 2 were utilized in the present analyses.

For score validation purposes, and to coincide with the clinician assessment, the following paper-pen questionnaires were completed by subjects at the Baseline visit (prior to seeing the clinician), and included in the analysis: the Inflammatory Bowel Disease Questionnaire (IBDQ) [12] and the EuroQol Five-dimension Questionnaire Three-level Version (EQ-5D-3 L) [13]. In addition, the score for patient-rated overall change in CD symptoms at Week 2 was utilized in the present analyses.

Clinicians completed the CDAI, with the baseline assessment used for these analyses. In addition, a single-item clinician-completed global rating of change (7-point scale, ranging from “very much worse” to “very much better”) in disease severity at Week 2 was included in these analyses.

#### Statistical analysis

Analyses were performed in accordance with a pre-specified statistical analysis plan. All statistical tests used a significance level of 0.05 (two-sided) unless otherwise noted. Statistical tests were adjusted for multiple comparisons as appropriate, using the Sheffe method. SAS version 9.2 was used for all statistical analyses, excepting the confirmatory factor analysis conducted with Mplus [14], and the Rasch analysis conducted using RUMM2030 [15].

Item-level analyses were calculated using single-day scores selected at random in the seven-day period leading up to the baseline study visit. These analyses included measures of central tendency, floor and ceiling effects, and inter-item correlations. An item was flagged for potential problems if it showed a floor (minimum response > 25%) or ceiling effect (maximum response > 25%), or when the inter-item correlation was greater than 0.80. Factor analyses were performed to evaluate the structure of the measure and develop a scoring algorithm. Approximation of simple structure with factor loadings greater than 0.30 were the criterion for accepting a factor solution; oblique rotation was allowed. Rasch analyses were conducted separately for each factor that consisted of a single dimension; items with negative fit residual value ≤ − 3.0 or positive fit residual ≥3.0 were flagged for potential deletion [16]. Factor and Rasch analyses were conducted using single-day scores selected at random using SAS version 9.2 from the week leading up to the Week 2 visit.

Once the items and scales were finalized, scores were tested for reliability and validity using Baseline and/or Week 2 data. Specifically, internal consistency reliability was assessed using Cronbach’s alpha coefficient, with a target value of 0.7 indicating good internal consistency [17, 18]. Test-retest reliability was assessed among those who reported relative stability in their condition over a two-week period (Baseline to Week 2) in two different ways. In the first analysis, test-retest reliability was assessed among subjects who reported relative stability in patient-reported global rating of change (i.e., “almost the same” or “about the same”). In the second analysis, test-retest reliability was assessed among those with no change based on a clinician-rated global raging of change in CD severity at Week 2. Intraclass correlation coefficients (ICC) were computed, whereby ≥0.7 indicates good test-retest reliability [18, 19].

Score validity was assessed by examining correlations of the CD-PRO/SS with the IBDQ scores and EQ-5D-3 L scores at Baseline using Spearman rank correlations. The CD-PRO/SS was expected to be moderately to highly correlated (> 0.40) [20] with IBDQ scores. No specific hypotheses were made with respect to the magnitude of the correlations between the CD-PRO/SS scores and the EQ-5D-3 L, although generally lower correlations were expected relative to those specified above, given that the EQ-5D-3 L is a generic health status measure as opposed to a specific measure of the signs and symptoms of CD.

Known-group validity was examined to determine whether the CD-PRO/SS could distinguish between patients by disease severity based on the patient-rated global assessment of disease severity, with mean CD-PRO/SS scores at Baseline compared by patient global scores categorized by “not at all” vs. “very mild,” “mild” vs. “moderate,” and “severe” vs. “very severe.” Analysis of covariance models with baseline clinical measurement groups as the main effects in the model were used.

## Results

### Study samples

Demographics and clinical characteristics for the qualitative studies are shown in Table 1. The study samples ranged in age from 19 to 72, representing a range in terms of ethnicity, race, extent of disease, and disease severity.

**Table 1:** Patient Demographic and Clinical Characteristics: Phase I, Qualitative

Demographic and Clinical Characteristics	Total (*n* = 49)
Age	
Mean (SD)	43.3 (15.43)
Range	(19–72)
Gender, n (%)	
Male	15 (30.6%)
Female	34 (69.4%)
Ethnicity, n (%)	
Hispanic or Latino	2 (4.1%)
Not Hispanic or Latino	46 (93.9%)
Missing	1 (2.0%)
Race,^a^ n (%)	
American Indian or Alaska Native	0 (0.0%)
Asian	1 (2.0%)
Black or African American	5 (10.2%)
Native Hawaiian or other Pacific Islander	0 (0.0%)
White	40 (81.6%)
Other	3 (6.1%)
SeCDAI score	
Mean (SD)	470.8 (262.82)
Range	(42–1078)
Remission (< 150)	5 (10.2%)
Mild (150–220)	3 (6.1%)
Moderate (≥ 220)	41 (83.7%)
Extent of disease, n (%)	
Affecting only the colon (large intestine)	14 (28.6%)
Affecting only the lower small intestine (ileum and jejunum)	6 (12.2%)
Affecting only the upper GI tract (stomach, esophagus, and duodenum)	0 (0.0%)
Affecting the colon (large intestine) and lower small intestine	22 (44.9%)
Affecting the lower small intestine and upper GI tract	3 (6.1%)
Affecting the colon (large intestine), and the lower small intestine, and the upper GI tract (stomach, esophagus, and duodenum)	4 (8.2%)
Disease location,^b^ n (%)	
L1, ileal only	4 (13%)
L2, colonic only	13 (43%)
L3, ileocolonic	12 (40%)
L4, isolated upper disease	–
Missing	1 (3%)
Disease behavior,^b,c^ n (%)	
B1, non-stricturing non-penetrating	14 (47%)
B2, stricturine	11 (37%)
B3, penetrating (not including abscess)	6 (20%)

Note: Percents may not add to 100 due to rounding

^a^Not mutually exclusive

^b^Data were only collected for additional concept elicitation and cognitive interview (*n* = 30)

^c^Responses are not mutually exclusive

*Abbreviations*: *n* Number, *SD* Standard deviation, *SeCDAI* Sandler estimated Crohn’s disease Activity Index

### Phase I: Development and content validity

Findings from focus groups and individual interviews identified nine sign and symptom items covering bowel and abdominal symptoms. Important bowel-related symptoms from the perspective of the patient included frequency, consistency, the presence of blood, and the urge/need to have a bowel movement (BM) right away. Key abdominal symptoms included pain in the stomach area, bloating, gas, nausea, and vomiting. The symptoms most relevant during flare-ups included frequency of and consistency of BMs, and pain in the stomach area. Patient descriptions of the symptoms they experienced during a flare were similar to the language they used to describe their everyday symptoms, just more severe and/or persistent. Patient descriptions of their symptom experience underline the variability not only within, but also between patients.

Additional details of the qualitative methods and results, along with evidence of saturation, are shown in the online Additional file 1.

The final version, ready for quantitative testing, was a daily diary comprising nine candidate symptom items covering all GI signs and symptoms identified by patients and confirmed by clinicians as relevant and important to the assessment of disease activity in CD. For number and consistency of bowel movements, response options were based on frequency. The number of bowel movements was queried on a 8-point scale with ranges considered reasonable and meaningful to patients and clinicians (0, 1–2, 3–4, 5–6, 7–9, 10–12, 13–17, 18–24, more than 24). The intent was to use quantitative data to evaluate these categories, with the possibility of combining and/or deleting categories, while maintaining a clinically meaningful and sensitive indicator of bowel movement frequency. For all other symptoms, response options were based on presence (yes/no) and severity or frequency of each, with scores ranging from 0 (none or not at all) to 4 (always or very severe); scores for the item “vomiting” ranged from 0 times to 4 or more times.

### Phase II: Score reliability and validity

#### Item and factor analysis and scoring algorithm

Item-by-item descriptive statistics are reported in Table 2. Subjects used the full range of response options for each of the items, with the exception of the item concerning number of bowel movements; no study participants reported > 24 bowel movements for the single-day scores selected at random. Four of nine items had a floor effect exceeding 25%, with more than 65% of patients reporting no vomiting (97.1%), no blood in bowel movement (76.5%), and no nausea (60.9%). Ceiling effects (> 25%) were considered high for only the number of liquid bowel movements (38.2%).

**Table 2:** Item Descriptive Characteristics CD-PRO/SS^a^ (*n* = 238)

CD Signs and Symptoms	Mean (SD)	Range	Floor, N (%)	Ceiling, N (%)
Number of bowel movements	4.1 (1.47)	1–8	7 (2.9%)	0 (0.0%)
Number of liquid bowel movements	2.8 (1.28)	0–4	21 (8.8%)	91 (38.2%)
Blood in bowel movements	0.6 (1.18)	0–4	182 (76.5%)	11 (4.6%)
Passing gas	2.0 (1.23)	0–4	41 (17.2%)	23 (9.7%)
Vomiting	0.1 (0.39)	0–4	231 (97.1%)	1 (0.4%)
Pain in belly	2.1 (1.16)	0–4	32 (13.4%)	26 (10.9%)
Feel nauseated	0.9 (1.21)	0–4	145 (60.9%)	7 (2.9%)
Bloating	1.7 (1.30)	0–4	71 (29.8%)	18 (7.6%)
Need to have bowel movement right away	2.2 (1.36)	0–4	49 (20.6%)	45 (18.9%)

^a^CD-PRO single-day item score which is selected at random from the non-missing days in the week preceding baseline visit.*Abbreviations*: *CD-PRO/SS* Crohn’s disease patient-reported outcome symptoms and signs, *n* Number

Due to the notable floor effects, items assessing the concepts of vomiting, blood in bowel movement, and nausea were deleted from the exploratory factor analysis model. After these three items were deleted, the findings support a two-factor solution (Comparative Fit Index = 0.98, Standardized Root Mean Square Residual = 0.033, and Root Mean Square of Approximation = 0.094).

One factor represents “Bowel Signs and Symptoms” and includes three items (number of BMs, BMs mostly or completely liquid, and urge to have BMs right away), while the other factor represents “Abdominal Symptoms” and also includes three items (pain in belly, bloating, and pass gas). Rasch analysis indicated that all of the fit residuals for items in each of the two models fell within the acceptable range (≥ − 3.0 and ≤ 3.0); however, several of the response categories were not ordered correctly, primarily due to very few responses for “rarely” and “mild” categories.

Taking into consideration findings from both the qualitative and quantitative studies, several decisions were reached regarding the CD-PRO/SS. First, given that few subjects endorsed the response category “more than 24” for the item “number of BMs,” this item response level was removed. As noted previously, three items (vomiting, blood in bowel movement, and nausea) were deleted due to high floor effects. Finally, although the Rasch analyses suggested the number of response options for several items could be reduced from a 5- to a 4-point scale by combining responses, the distinction between “none” and “mild” and between “mild” and “moderate” was considered clinically important and the decision was made to retain the 5-point scaling.

The final CD-PRO/SS assesses two important indicators of disease activity in CD: Bowel Signs and Symptoms (three items) and Abdominal Symptoms (three items), with each scored as a simple mean across all items comprising the scale. There is no single total score that combines both scales.

#### Reliability

Adequate internal consistency reliability was demonstrated with alpha coefficients of 0.74 for Bowel Signs and Symptoms and 0.67 for Abdominal Symptoms. Although findings indicate that the Cronbach’s alpha for the domain of Abdominal Symptoms would increase slightly to 0.69 with the deletion of “passing gas,” the item was retained based on importance of this symptom from the patient perspective. Test-retest reliability in stable patients was supported by both patient-reported change in symptom (*n* = 110) and clinician-reported change in symptom severity (*n* = 126) over a two-week period, with ICC values > 0.80 for both scales.

#### Validity

Correlations between the CD-PRO/SS domain scores and other relevant PRO measures are presented in Table 3. All relationships were confirmed based on a priori predictions, with CD-PRO/SS Bowel Signs and Symptoms domain score demonstrating moderate correlations with IBDQ bowel frequency item (*r* = 0.43), and the CD-PRO/SS Abdominal Scale domain score demonstrating moderate correlations with the IBDQ bloating items (*r* = 0.54) and the IBDQ bowel system score (*r* = 0.48). As anticipated, weaker correlations were observed between the CD-PRO/SS scales with measures of IBDQ emotional health and social functions scores (discriminant validity) and the EQ-5D-3 L scores.

**Table 3:** Construct Validity: CD-PRO/SS Score Correlations^a^ with IBDQ Scores at Baseline

CD-PRO/SS	IBDQ Item and Scale Scores						
	Item 1 (Bowel Frequency)	Item 20 (Bloating)	Item 22 (Rectal Bleeding)	Bowel System	Systemic System	Emotion Health	Social Function
BM Scale	.43	.06	.08	.26	.09	.10	.19
Number of bowel movements	.37	.15	.07	.14	.02	.03	.15
Mostly liquid bowel movements	.32	.08	.01	.16	.14	.02	.10
Need to have bowel movement right away	.35	.04	.10	.29	.09	.15	.14
Abdominal Scale	.15	.54	.02	.48	.27	.26	.24
Pass gas	.17	.26	.06	.30	.10	.20	.13
Pain in belly	.14	.25	.06	.38	.30	.15	.23
Bloating	.07	.69	.01	.43	.26	.27	.20

^a^Spearman’s correlation coefficient

*Abbreviations*: *CD-PRO/SS* Crohn’s disease patient-reported outcome signs and symptoms, *IBDQ* Inflammatory Bowel Disease Questionnaire, *p p*-value, *r* Rating

The Bowel Signs and Symptoms and Abdominal Symptoms Scales were each able to significantly differentiate among the moderate and severe groups as defined by patient ratings of their symptom severity (*p* < 0.05). Pairwise comparisons that included the mild and very severe disease severity groups were not statistically significantly different for either of the CD-PRO/SS scales.

## Discussion

The CD-PRO/SS measure was developed to standardize the quantification of GI signs and symptoms of CD in clinical trials through direct patient ratings. The methodology used to develop the CD-PRO/SS followed the US FDA Guidance on PRO instrument development, which conveys the agency’s thinking on best practices for the development of measures and the evidence needed for the agency’s evaluation [7]. The position of the US FDA is that the CDAI is no longer an acceptable measure to assess the signs and symptoms of CD for labeling purposes and that these concepts are best measured, scored, and reported independently, separating clinical findings from patient-reported symptoms, with the latter coming directly from the patients themselves using a reliable and valid measure fit for this purpose [7, 21–23]. The European Medicines Agency also discourages the use of the CDAI as a primary endpoint for future clinical studies, and instead recommends that signs, symptoms, and inflammation be evaluated independently [24]. While items derived from the CDAI (including stool frequency and abdominal pain) are currently being used in clinical trials, these PRO measures are meant for interim use only to ensure that new drug therapies can progress [25, 26]. The CD-PRO/SS represents a new measure to evaluate treatment-related outcomes from the perspective of the patient and to support labeling claims related to the GI signs and symptoms of CD in clinical trials of adults (age 18 or older) with moderate-to-severe CD treated in outpatient settings.

The CD-PRO/SS was developed based on data collected from focus groups and one-to-one concept elicitation interviews, input from clinical experts, and refined through a process of cognitive interviews, all with representatives of the target population. Every effort was made to ensure that the qualitative phase of research was conducted in a manner that was conclusive with respect to concept elicitation and that sufficient cognitive interviews were conducted to ensure that comprehension and readability were acceptable. Measurement properties were tested based on a secondary analysis of clinical trial data of 238 adults with moderate-to-severe CD.

The decision to retain or delete items for the final measure was an iterative process with consideration of floor and ceiling effects, results from the factor and Rasch analyses, previous qualitative results, and clinical considerations. Several items included in the Bowel Signs and Symptoms and Abdominal Symptoms Scales had high floor effects (> 30%), most notably vomiting, blood in bowel movement, and nausea, and were subsequently deleted from the CD-PRO/SS. Results of the Rasch analyses of response options suggests that there is little response distinction between “none” and “mild” or between “never” and “rarely.” These responses were retained, however, given the importance of the response distinction from a clinical perspective, to capture degrees of improvement in more severe patients, with the understanding that further evaluation will be needed to confirm their suitability and utility across populations.

The final CD-PRO/SS includes two scales: Bowel Signs and Symptoms (three items) and Abdominal Symptoms (three items), with both scales scored separately. Performance testing of the CD-PRO/SS scores demonstrated evidence of internal consistency and reproducibility. The CD-PRO/SS scores showed moderate correlations with other relevant measures identified a priori. The CD-PRO/SS scores also appear to have known-group validity with significant differences in both domain scores between moderate and severe disease groups when defined by patient global ratings of disease severity. Pairwise comparisons that included the mild and very severe severity groups were not statistically significantly different, due in large part to the small sample size in the mild (*n* = 7) and very severe (*n* = 20) groups at baseline.

Both scales of the CD-PRO/SS include multiple items to better capture the bowel and abdominal symptom experience of CD from the perspective of the patient, which allows for a more granular assessment of aspects of the disease that are relevant and important to patients. In clinical trials of therapies for CD, the CD-PRO/SS potentially can provide data for a co-primary endpoint or a key secondary endpoint. Therapies targeting inflammation in induction studies could use an objective marker of inflammation (e.g., endoscopy, magnetic resonance enterography, fecal calprotectin) to assess the co-primary or primary endpoint, with the Bowel Signs and Symptoms module as the assessment of a co-primary or key secondary endpoint. Therapies expected to improve functional abdominal symptoms might use this module as the primary endpoint, while maintenance studies of anti-inflammatory studies might use a co-primary endpoint of an objective marker of inflammation and the Bowel Signs and Symptoms and Abdominal Symptoms scales to demonstrate a long-term significant impact on multiple symptom domains important to patients.

Several limitations should be noted for this research. First, the sample included a predominance of patients with moderate-to-severe CD, due to inclusion criteria for the clinical study which required patients to have a CDAI score ≥ 220 and ≤ 450. In general, CD clinical trials enroll patients whose disease severity is moderate to severe at baseline disease severity, so as to be able to evaluate improvement during the entire treatment period. While these patients represent the target population for the final CD-PRO/SS, it is generally best to include subjects with a full range of disease severity in psychometric evaluation studies to optimize testing and assure consistent score performance across this overall range in severity. In addition, only two weeks of clinical trial data were available for analysis, precluding the evaluation of responsiveness to change in clinical status.

In conclusion, the CD-PRO/SS is a new daily diary to gather data on the gastrointestinal signs and symptoms of CD directly from the patient. The instrument was developed to meet regulatory guidance, with initial validation evidence suggesting that the CD-PRO/SS scores are reliable, valid, and ready for use and further testing in clinical trials. The CD-PRO/SS complements and extends information provided by the clinician, endoscopy, and biomarkers in clinical studies.

## Additional file

Additional file 1: Development and validation of the Crohn’s Disease Patient-reported Outcomes Signs and Symptoms (CD-PRO/SS) Diary. (DOCX 117 kb)

## Abbreviations

BM: Bowel movement
CD: Crohn’s disease
CDAI: Crohn’s Disease Activity Index
CD-PRO: Crohn’s Disease Patient-reported Outcomes
CD-PRO/SS: Crohn’s Disease Patient-reported Outcomes Signs and Symptoms
EQ-5D-3 L: EuroQol Five-dimension Questionnaire Three-level Version
FDA: Food and Drug Administration
GI: Gastrointestinal
IBD: Inflammatory bowel disease
IBDQ: Inflammatory Bowel Disease Questionnaire
ICC: Intraclass correlation coefficients
IRB: Institutional review board
PRO: Patient-reported outcomes
SD: Standard deviation
SeCDAI: Sandler estimated Crohn’s disease Activity Index
SS: Signs and symptoms
TNF: Anti-tumor necrosis factor
US: United States
