# Supplementary material for: Development and validation of the Crohn’s disease patient-reported outcomes signs and symptoms (CD-PRO/SS) diary
Source: J Patient Rep Outcomes. 2018 May 9;2:24. doi: 10.1186/s41687-018-0044-7 (PMC5942337; doi:10.1186/s41687-018-0044-7)
Supplement: Supplementary file 1 — Development and validation of the Crohn’s Disease Patient-reported Outcomes Signs and Symptoms (CD-PRO/SS) Diary. (DOCX 117 kb) [file 41687_2018_44_MOESM1_ESM.docx]

**Title:** Development and Validation of the Crohn’s Disease Patient-reported Outcomes Signs and Symptoms (CD-PRO/SS) Diary

# Additional file 1

## Phase I: Qualitative Research for the Development and Content Validity of the CD-PRO Signs and Symptoms (CD-PRO/SS) Measure and Systemic Symptom Scales

Initial development of the Crohn’s Disease Patient-reported Signs and Symptoms Scale (CD-PRO/SS) and Systemic Symptoms Scale are based on qualitative work conducted by Dr. Peter Higgins at the University of Michigan Health Systems during 2009–2010. The IBD PRO Consortium was formed in early 2011 to develop PRO drug development tools for clinical trials evaluating treatment efficacy of IBD, specifically CD and ulcerative colitis. The early work of Dr. Higgins and data from the focus groups were provided to the Consortium. Additional one-to-one concept elicitation interviews and cognitive interviews were performed by Evidera (formerly part of United BioSource Corporation) under the direction of the IBD PRO Consortium. Findings from the focus group discussions and concept elicitation interviews were used to generate items that formed the foundation of the initial CD-PRO/SS and Systemic Symptoms Scale.

The development of the CD-PRO/SS and Systemic Symptoms scales are based on qualitative studies conducted among a total of 49 patients with Crohn’s Disease (CD). To determine instrument content and structure, 29 patients with moderate-to-severe CD participated in concept elicitation focus groups (Phase I) or one-to-one qualitative interviews (Phase II). The focus group discussions provided the opportunity to initially explore the important underlying concepts related to symptom experience and the language patients use to describe their condition. Concept elicitation interviews were conducted to obtain in-depth elicitation of symptoms, evaluate saturation of concepts, and obtain additional, expanded information with respect to patient language, variability in symptom experience, and impact on health-related quality of life when symptoms worsen or improve with treatment. Findings from focus group and individual interview-based concept elicitation methods were used to generate an item pool, inform response options, and determine appropriate recall. Two rounds of cognitive interviews were subsequently conducted among 20 patients with moderate-to-severe CD to further support the saturation and relevancy of items, as well as refine the measure to ensure clarity and understanding among the target patient population.

All interviews were audio-recorded and transcribed. Content analyses were performed by independent coders, with data organized in NVivo or ATLAS.ti.

### Focus Groups

A total of 20 patients with CD participated in one of five focus groups. Patients were recruited from five clinical sites located at the University of Michigan, University of North Carolina Chapel Hill, University of California San Diego, University of Maryland, and Alameda County Medical Center. Patients with CD were between the ages of 18 and 75 years. Target enrollment included at least 75% of participants with a period of at least **moderate** disease activity using the Sandler estimated CD Activity Index (SeCDAI > 220) and at least 25% of participants with at least **severe** activity (SeCDAI > 450) during the past year; scores for participants were n=1 with SeCDAI 220–450, and n=19 with a SeCDAI >450. Experienced scientific staff from the University of Michigan moderated all focus groups using a semi-structured discussion guide to ensure consistency across all focus group discussions. The guide was designed to elicit information about the patients’ CD signs and symptom experiences as they worsened during a flare of their condition.

A total of 11 concepts emerged in the first focus group, with additional concepts decreasing rapidly in the subsequent four focus group discussions (number of new concepts emerging totaled 9, 3, 3, and 2 for focus groups 2–5, respectively).

Symptoms reported by more than half of the participants included [joint pain/general body pain](file:///\\evidera.com\ProjectFiles\Production\A-13029\CD%20Briefing%20Document\Final\Appendices\G_A-11980%20Crohn's%20Disease%20CE%20Report_rev09Dec13_Final_clean.pdf), consistency of bowel movement, frequency of bowel movement, [pain in the stomach area](file:///\\evidera.com\ProjectFiles\Production\A-13029\CD%20Briefing%20Document\Final\Appendices\G_A-11980%20Crohn's%20Disease%20CE%20Report_rev09Dec13_Final_clean.pdf), and [weight change](file:///\\evidera.com\ProjectFiles\Production\A-13029\CD%20Briefing%20Document\Final\Appendices\G_A-11980%20Crohn's%20Disease%20CE%20Report_rev09Dec13_Final_clean.pdf).

The most frequently reported symptom that worsened during a flare among participants was joint pain/general body pain (80%). Participants who experienced pain in their joints described this pain as *“joint pain,” “inflammation,”* and *“swelling.”* These participants also indicated that this pain was specific to their *“knees,” “knuckles,”* and *“elbows*.*”* Several subjects (n=3) also reported experiencing general body pain, pain in the “*lower* *back,”* pain in the *“stricture*,*”* or feeling *“uncomfortable.”* The description of pain severity and duration varied by participant and included *“exquisite pain,” “debilitating,” “comes and goes,” “severe pain,”* *“sharp pain,”* and hurting “*all the time*.*”*

Participants also mentioned consistency and frequency of BM as an important symptom in all five focus groups. Participants described stool consistency as being, *“loose,”* *“diarrhea,”* and *“liquid.”* With respect to frequency of BMs, several participants reported a specific number of BMs ranging from “*10–15 times a day*” (n=1) to “*15–20 times a day”* (n=4), while others mentioned general increases (n=8) or decreases (n=1) in BM frequency throughout the day or week, depending on the participant, course of treatment, and occurrence of a flare. Participants described their stomach area pain as a *“really bad stomach ache,” “stomach cramp,*” or “*cramping.*” The type, onset, and location of pain in the stomach area varied by individual, but the common themes among participants were intensity of the stomach pain (including cramping), feeling worse during and after eating, having to use the bathroom, and experiencing pain in the lower abdomen. Throughout each focus group discussion, participants characterized their weight change in terms of *“losing weight.”* Although participant experiences with weight loss varied, common themes emerged surrounding the severity of the weight loss and weight loss due to *“flaring.”* Other important symptoms included [blood in BM](file:///\\evidera.com\ProjectFiles\Production\A-13029\CD%20Briefing%20Document\Final\Appendices\G_A-11980%20Crohn's%20Disease%20CE%20Report_rev09Dec13_Final_clean.pdf), [urge/need to have a BM right away](file:///\\evidera.com\ProjectFiles\Production\A-13029\CD%20Briefing%20Document\Final\Appendices\G_A-11980%20Crohn's%20Disease%20CE%20Report_rev09Dec13_Final_clean.pdf), [gas](file:///\\evidera.com\ProjectFiles\Production\A-13029\CD%20Briefing%20Document\Final\Appendices\G_A-11980%20Crohn's%20Disease%20CE%20Report_rev09Dec13_Final_clean.pdf), [nausea](file:///\\evidera.com\ProjectFiles\Production\A-13029\CD%20Briefing%20Document\Final\Appendices\G_A-11980%20Crohn's%20Disease%20CE%20Report_rev09Dec13_Final_clean.pdf), [weakness](file:///\\evidera.com\ProjectFiles\Production\A-13029\CD%20Briefing%20Document\Final\Appendices\G_A-11980%20Crohn's%20Disease%20CE%20Report_rev09Dec13_Final_clean.pdf), [tiredness](file:///\\evidera.com\ProjectFiles\Production\A-13029\CD%20Briefing%20Document\Final\Appendices\G_A-11980%20Crohn's%20Disease%20CE%20Report_rev09Dec13_Final_clean.pdf), [bloating](file:///\\evidera.com\ProjectFiles\Production\A-13029\CD%20Briefing%20Document\Final\Appendices\G_A-11980%20Crohn's%20Disease%20CE%20Report_rev09Dec13_Final_clean.pdf), [dehydration](file:///\\evidera.com\ProjectFiles\Production\A-13029\CD%20Briefing%20Document\Final\Appendices\G_A-11980%20Crohn's%20Disease%20CE%20Report_rev09Dec13_Final_clean.pdf), [lack of appetite](file:///\\evidera.com\ProjectFiles\Production\A-13029\CD%20Briefing%20Document\Final\Appendices\G_A-11980%20Crohn's%20Disease%20CE%20Report_rev09Dec13_Final_clean.pdf), and [vomiting](file:///\\evidera.com\ProjectFiles\Production\A-13029\CD%20Briefing%20Document\Final\Appendices\G_A-11980%20Crohn's%20Disease%20CE%20Report_rev09Dec13_Final_clean.pdf).

### Qualitative Interviews

While focus groups allow for a dynamic interchange between participants which often leads to a broader range of information discussed, patient language and opinions may become influenced by other group members. Individual interviews, on the other hand, provide an opportunity to explore in more depth concepts elicited during the focus group exchange, as well as an opportunity to more accurately track important underlying concepts obtained spontaneously by individuals. Thus, to ensure saturation on important signs and symptoms of CD from the perspective of the patient and extend results of Phase I, nine one-to-one qualitative interviews were conducted with adults with CD, recruited from two GI clinics, one in Poughkeepsie, NY, and the other in Torrance, CA. Participants had been diagnosed with CD for at least six months prior to screening; six participants had at least moderate disease activity based on a SeCDAI score of ≥220, with three participants in remission (SeCDAI <150); those with an ileostomy or colostomy in the previous four months were excluded. It should be noted that although biopsy was not initially included as eligibility criteria, biopsy confirmation of CD was obtained from the clinical sites for the nine interviewed participants, per the request of the FDA.

All interviews were conducted using a semi-standardized interview guide to ensure consistency across all subjects. The purpose of the interviews was three-fold: 1) identify important CD symptoms obtained spontaneously from patients using open-ended probes; 2) further explore the frequency and variability of symptom experience as identified by the patient; and 3) inform the development of response options and appropriate recall of the symptom measure. Throughout the interviews, the interviewer listened for terms and wording spontaneously voiced by participants as they described their symptoms. Discussion focused on participants’ current experiences, their experiences during an episode or flare-up, and the impact of these symptoms on their daily life.

Similar to findings from the focus groups, important symptoms were consistency of bowel movement, [tiredness](file:///\\evidera.com\ProjectFiles\Production\A-13029\CD%20Briefing%20Document\Final\Appendices\G_A-11980%20Crohn's%20Disease%20CE%20Report_rev09Dec13_Final_clean.pdf), [weakness](file:///\\evidera.com\ProjectFiles\Production\A-13029\CD%20Briefing%20Document\Final\Appendices\G_A-11980%20Crohn's%20Disease%20CE%20Report_rev09Dec13_Final_clean.pdf), [nausea](file:///\\evidera.com\ProjectFiles\Production\A-13029\CD%20Briefing%20Document\Final\Appendices\G_A-11980%20Crohn's%20Disease%20CE%20Report_rev09Dec13_Final_clean.pdf), [vomiting](file:///\\evidera.com\ProjectFiles\Production\A-13029\CD%20Briefing%20Document\Final\Appendices\G_A-11980%20Crohn's%20Disease%20CE%20Report_rev09Dec13_Final_clean.pdf), [weight loss](file:///\\evidera.com\ProjectFiles\Production\A-13029\CD%20Briefing%20Document\Final\Appendices\G_A-11980%20Crohn's%20Disease%20CE%20Report_rev09Dec13_Final_clean.pdf), [joint pain/general body pain](file:///\\evidera.com\ProjectFiles\Production\A-13029\CD%20Briefing%20Document\Final\Appendices\G_A-11980%20Crohn's%20Disease%20CE%20Report_rev09Dec13_Final_clean.pdf), frequency of bowel movement, [gas](file:///\\evidera.com\ProjectFiles\Production\A-13029\CD%20Briefing%20Document\Final\Appendices\G_A-11980%20Crohn's%20Disease%20CE%20Report_rev09Dec13_Final_clean.pdf), [urge/need to have BM right away](file:///\\evidera.com\ProjectFiles\Production\A-13029\CD%20Briefing%20Document\Final\Appendices\G_A-11980%20Crohn's%20Disease%20CE%20Report_rev09Dec13_Final_clean.pdf), [bloating](file:///\\evidera.com\ProjectFiles\Production\A-13029\CD%20Briefing%20Document\Final\Appendices\G_A-11980%20Crohn's%20Disease%20CE%20Report_rev09Dec13_Final_clean.pdf), [loss of appetite](file:///\\evidera.com\ProjectFiles\Production\A-13029\CD%20Briefing%20Document\Final\Appendices\G_A-11980%20Crohn's%20Disease%20CE%20Report_rev09Dec13_Final_clean.pdf), and blood in bowel movement.

The symptoms that were most relevant during episodes of flare included [pain in stomach area](file:///\\evidera.com\ProjectFiles\Production\A-13029\CD%20Briefing%20Document\Final\Appendices\G_A-11980%20Crohn's%20Disease%20CE%20Report_rev09Dec13_Final_clean.pdf), [consistency of bowel movements](file:///\\evidera.com\ProjectFiles\Production\A-13029\CD%20Briefing%20Document\Final\Appendices\G_A-11980%20Crohn's%20Disease%20CE%20Report_rev09Dec13_Final_clean.pdf), [weakness](file:///\\evidera.com\ProjectFiles\Production\A-13029\CD%20Briefing%20Document\Final\Appendices\G_A-11980%20Crohn's%20Disease%20CE%20Report_rev09Dec13_Final_clean.pdf), frequency of bowel movement, and [nausea](file:///\\evidera.com\ProjectFiles\Production\A-13029\CD%20Briefing%20Document\Final\Appendices\G_A-11980%20Crohn's%20Disease%20CE%20Report_rev09Dec13_Final_clean.pdf), also consistent with findings from the focus group discussions. Patient descriptions of the symptoms they experienced during a flare were similar to language that they used to describe their everyday symptoms, just more severe and/or persistent. In addition, patients noted that the severity of a symptom could vary during a flare episode within the day (particularly with respect to pain), or day to day. Patient descriptions of their symptom experience underline the variability not only within, but also between patients.

Findings from the focus group discussions and one-to-one qualitative interviews indicated that data were gathered to the point of saturation (Figure S1), with comprehensive results obtained by using the two elicitation methods.

Figure S1. Saturation of Concepts

Abbreviations: BM = bowel movement

### Development of Rating Scale

Based on findings from the focus group discussions, qualitative interviews, and input from clinical experts, a list of relevant items was generated which formed the foundation of the draft of CD-PRO/SS and Systemic Symptoms scales. Instructions, item stems, and response options were derived from patient language elicited during focus groups and interviews to ensure appropriateness, relevance, understanding, and clarity of the items included in the measure. A list of the symptom items and rationale for item wording based on focus group discussion, concept elicitation interviews, and cognitive interviews is included in Table S1.

Table S1. Symptom Items and Rationale for Wording

| Symptom Items | Rationale for Wording |
| --- | --- |
| Number of bowel movements | - Patient language - Use “bowel movement” instead of “stool” to describe number of times going to the toilet (and including “loose, watery stools” and “diarrhea”) |
| Bowel movements mostly or completely liquid (frequency) | - Based on patient descriptions of “diarrhea,” “runs,” “loose watery stools” and other similar expressions |
| Blood in your bowel movements (frequency) | - Patient language |
| Vomit (frequency) | - Patient language |
| Nausea (severity) | - Patient language |
| Passing gas (frequency) | - Patient language |
| Feel the need to have a bowel movement right away (severity) | - Based on patient descriptions of “urgency,” “urge to go right away,” and other similar expressions |
| Pain in your belly (severity) | - Capture pain in stomach area - Based on patient descriptions of pain in “stomach,” “lower intestine,” “upper intestine,” “abdomen” |
| Feel bloating in your belly (severity) | - Patient language “bloating” - “Belly” used to describe stomach area |
| Feel pain in your knees, hips, and/or elbows (severity) | - Based on patient descriptions of general body pain and joint pain (not specific to stomach area) - Excludes joints commonly associated with arthritic pain |
| Feel tired (severity) | - Patient language |
| Lack an appetite (severity) | - Patient language |
| Feel weak (severity) | - Patient language |
| Feel thirsty (severity) | - Clarification of patient term “dehydration” |

Data from the psychometric validation study, based on a secondary analysis of clinical trial data, were used to inform scoring and evaluate the psychometric properties of CD-PRO/SS domains (Bowel Movement Signs and Symptoms and Abdominal Symptoms) and Systemic Symptoms scale.

### Cognitive Interviews and Modifications

Two rounds of one-to-one interviews were conducted with CD patients (N=20) with confirmed diagnosis based on available biopsy, using cognitive interview methodology, examining readability and comprehensiveness. In each case, the cognitive interview followed the participant’s completion of the questionnaire and used a standardized discussion guide. For both rounds of cognitive interviews, subjects were first asked if there were other symptoms they experienced that were not captured on the questionnaire to confirm concept saturation. Subjects were then cognitively interviewed to establish: 1) the clarity of the items; 2) how the respondents interpret the items and how well their understanding matches the concept purportedly measured by the item; 3) ease of completion of the items; 4) the comprehensiveness of the instrument; and 5) the appropriateness of the format, response scales, and recall period. Eligibility criteria were the same as that used in the one-to-one concept elicitation qualitative interviews. All interviews were audio-recorded and transcribed. Content analyses were performed by independent coders, with data organized in ATLAS.ti.

The first round of cognitive interviews included 16 patients with CD recruited through three clinical sites specializing in gastroenterology, located in Poughkeepsie, NY; Torrance, CA; and Charlotte, NC. No new concepts emerged during the interviews. Overall, participants reported that the pen-paper version of the CD-PRO/SS easy to complete and understand, with items relevant to the symptoms that they experience. The instructions, item content, and response scales were clear and well-understood, with a few exceptions. For the symptoms blood in BM, vomit, and passing gas, it was unclear how patients were interpreting the response options related to symptom severity. With respect to recall, patients felt that daily recall period (past 24 hours) was appropriate and realistic for rating the presence and severity of this and other CD symptoms. Based on findings, the following modifications were made:

- Blood in bowel movement: Response options revised from severity to frequency (yes/no, rarely, sometimes, often, always) to better reflect patient experience; instructions modified accordingly.
- Vomit: Response options revised from severity to frequency (1 Time, 2 Times, 3 Times, 4 or more Times); instructions modified accordingly.
- Passing gas: Response options revised from severity to frequency (yes/no, rarely, sometimes, often, very often) to better reflect patient experience; instructions modified accordingly.
- Bowel movements mostly or completely liquid: Response option “not at all” revised to “never.” Although subjects did not indicate a problem with this response category, “not at all” reflects severity and is inconsistent with other response categories that address frequency.
- Nausea, pain in belly, bloating in belly, need to have bowel movement right away, pain in knees, hips, and/or elbows: To address variability within a 24-hour period, instructions modified from “If Yes, how severe was this symptom” to “If Yes, how severe was this symptom at its worst.”
- For items with severity-based response options, the response option “very mild” was deleted, as it was felt that it would be difficult for patients to distinguish between “very mild” and “mild.”

The second round of four additional cognitive interviews was conducted to confirm changes and to further explore patient understanding and relevancy of the items and corresponding response options. Round 2 also provided an opportunity to examine patient understanding of the CD-PRO/SS and Systemic Symptoms scale administered electronically as a hand-held device. No new concepts emerged during the second round of cognitive interviews, with patients confirming that the CD-PRO/SS and Systemic Symptom scale captured all the relevant symptoms they experienced. Patients found the revised CD-PRO/SS and ePRO administration easy to complete and understand. Findings indicate that patients understood the items as intended. The recall period and response options were found to be appropriate. Based on findings from Round 2, no additional modifications were made the CD-PRO/SS.

The preliminary conceptual framework, based on qualitative data and clinical expert input, outlines a score for CD Signs and CD Symptoms, with the possibility of a combined score of CD signs and symptoms (Figure S2).

Figure S2. Preliminary Hypothesized Conceptual Framework Based on Qualitative Research

Frequency of BMs mostly or completely liquid

Number of BMs

Frequency of blood in BM

CD

Signs

Severity of nausea

Frequency of passing gas

Severity of need to have a BM right away

Severity of pain in belly

Severity of bloating in belly

CD

Symptoms

Severity of pain in knees, hips, and/or elbows

Severity of feeling tired

Severity of lack of appetite

Severity of feeling weak

Severity of feeling thirsty

Frequency of vomiting

Systemic Symptoms

CD

Signs and Symptoms

**General Concepts**

**Item**

**-**

**Level Concept**

Systemic Symptoms

Abbreviations: BM = bowel movement

## Phase II: Quantitative Research

Supplemental results from the quantitative analysis for the CD-PRO/SS and Systemic Symptoms Scales are provided in Tables S2–S10 and Figures S3-S4.

Table S2. Item Analysis: Systemic Symptoms Item Descriptive Characteristics at Baseline^1^

| **Systemic Symptoms** | **Baseline (N=238)** | | | | |
| --- | --- | --- | --- | --- | --- |
|  | **Mean (SD)** | **Median** | **Range** | **N (%) Floor** | **N (%) Ceiling** |
| Pain in knees, hips, and/or elbows | 1.3 (1.32) | 1 | 0–4 | 109 (45.8%) | 11 (4.6%) |
| Feel tired | 2.1 (1.23) | 2 | 0–4 | 38 (16.0%) | 32 (13.4%) |
| Feel weak | 1.6 (1.30) | 2 | 0–4 | 71 (29.8%) | 21 (8.8%) |
| Lack appetite | 0.9 (1.15) | 0 | 0–4 | 140 (58.8%) | 6 (2.5%) |
| Feel thirsty | 1.4 (1.30) | 1 | 0–4 | 92 (38.7%) | 14 (5.9%) |

^1^ Single-day item score which is selected at random from the non-missing days in the week preceding baseline visit.

Abbreviations: N = number; SD = standard deviation

Table S3. Exploratory Factor Analysis Factor Loadings Excluding Blood, Vomiting, and Nausea: CD-PRO/SS^1^

| **Item** | **Model Fit Statistics** | **Factor 1** | **Factor 2** | **Factor 3** |
| --- | --- | --- | --- | --- |
| Number of bowel movements | CFI = 0.979 | **0.736** | 0.047 | **--** |
| Mostly liquid bowel movements | SRMR = 0.033 | **0.615** | 0.119 | **--** |
| Passing gas | RMSEA = 0.094  (0.038, 0.156) | 0.028 | **0.530** | **--** |
| Pain in belly |  | 0.249 | **0.608** | **--** |
| Bloating |  | 0.148 | **0.821** | **--** |
| Need to have bowel movement right away |  | ***0.714*** | ***0.499*** | **--** |

^1^ Single-day item score which is selected at random from the non-missing days in the week preceding baseline visit.

Abbreviations: CD-PRO/SS = Crohn’s disease patient-reported outcome signs and symptoms; CFI = Comparative Fit Index; RMSEA = Root Mean Square Error of Approximation; SRMR = Standardized Root Mean Square Residual

Table S4. Confirmatory Factor Analyses: Systemic Symptoms Scale^1^

| **Systemic Symptoms Scale** | **Standardized Coefficient** | **Model Fit Statistics^2^** |
| --- | --- | --- |
| Pain in knees, hips, and/or elbows | **0.464** | CFI = 0.992 |
| Feel tired | **0.849** | RMSEA (95% CI) = 0.070 (0.000, 0.128) |
| Feel weak | **0.859** | WRMR = 0.466 |
| Lack appetite | **0.595** |  |
| Feel thirsty | **0.497** |  |

^1^ Single-day CD-PRO scores from week 2.

^2^ Fit criteria = CFI ≥0.90; RMSEA <0.08; WRMR < 1.0. RMSEA may be ignored if other fit statistics are good. Items with standardized coefficient <0.30 will be reviewed for possible deletion.

Abbreviations: CFI = Comparative Fit Index; RMSEA = Root Mean Square of Approximation; WRMR = Weighted Root Mean Square Residual

Table S5. Internal Consistency Reliability of CD-PRO/SS and Systemic Symptoms Scales

| **Domain/Item** | **N** | **Cronbach’s α** | **Cronbach’s α if Item Deleted** |
| --- | --- | --- | --- |
| **Bowel Signs and Symptoms** | **231** | **0.74** |  |
| Number of bowel movements |  |  | 0.65 |
| Mostly liquid bowel movements |  |  | 0.66 |
| Need to have bowel movement right away |  |  | 0.65 |
| **Abdominal Scale** | **231** | **0.68** |  |
| Passing gas |  |  | 0.69 |
| Pain in belly |  |  | 0.58 |
| Bloating |  |  | 0.46 |
| **Systemic Symptoms** | **231** | **0.77** |  |
| Pain in knees, hips, and/or elbows |  |  | 0.77 |
| Feel tired |  |  | 0.68 |
| Feel weak |  |  | 0.68 |
| Lack appetite |  |  | 0.75 |
| Feel thirsty |  |  | 0.75 |

Abbreviations: α = alpha; N = number

Table S6. Test-Retest Reliability among Stable Subjects Based on Patient Global Rating of Change^1^

| **Domain** | **N** | **Baseline Mean (SD)** | **Week 2 Mean (SD)** | **Difference^2^** | **ICC** |
| --- | --- | --- | --- | --- | --- |
| Bowel Signs and Symptoms | 110 | 2.6 (1.04) | 2.5 (1.02) | -0.08 | 0.84 |
| Abdominal Scale | 110 | 1.9 (0.86) | 1.8 (0.82) | -0.03 | 0.83 |
| Systemic Symptoms | 110 | 1.4 (0.81) | 1.3 (0.80) | -0.08 | 0.82 |

^1^ Stable subjects defined as patients that rated their change in condition on PGRC at week 2 as “worse, almost the same, not important”, “about same”, or “better, almost the same, not important”

^2^ Mean difference=Week 2 – Baseline

Abbreviations: ICC = intraclass correlation coefficient; N = number; SD = standard deviation

Table S7. Test-Retest Reliability of the CD-PRO/SS Domain Scores among Stable Subjects Based on Clinician Global Ratings of Change^1^

| **Domain** | **N** | **Baseline Mean (SD)** | **Week 2 Mean (SD)** | **Difference^1^** | **ICC** |
| --- | --- | --- | --- | --- | --- |
| Bowel Signs and Symptoms | 126 | 2.6 (0.99) | 2.6 (0.97) | -0.00 | 0.87 |
| Abdominal Scale | 126 | 1.9 (0.81) | 1.9 (0.81) | -0.06 | 0.82 |
| Systemic Symptoms | 126 | 1.3 (0.78) | 1.3 (0.78) | -0.03 | 0.84 |

^1^ Mean difference=Week 2 – Baseline

Abbreviations: CD-PRO/SS = Crohn’s disease patient-reported outcome signs and symptoms; ICC = intraclass correlation coefficient; N = number; SD = standard deviation

Table S8. Construct Validity: CD-PRO/SS and Systemic Symptoms Score Correlations^1^ with IBDQ Scores at Baseline

| **Domain** | **IBDQ Item and Scale Scores r (p)** | | | | | | |
| --- | --- | --- | --- | --- | --- | --- | --- |
|  | **Item 1 (Bowel Frequency)** | **Item 20 (Bloating)** | **Item 22 (Rectal Bleeding)** | **Bowel System** | **Systemic System** | **Emotion Health** | **Social Function** |
| **Bowel Signs and Symptoms** | .43 (<0.0001) | .06 (0.3643) | .08 (0.2037) | .26 (<0.0001) | .09 (0.1889) | .10 (0.1372) | .19 (0.0048) |
| Number of bowel movements | .37 (<.0001) | .15 (0.0234) | .07 (0.2909) | .14 (0.0321) | .02 (0.7406) | .03 (0.5999) | .15 (0.0204) |
| Mostly liquid bowel movements | .32 (<.0001) | .08 (0.2550) | .01 (0.9037) | .16 (0.0191) | .14 (0.0389) | .02 (0.7266) | .10 (0.1270) |
| Need to have bowel movement right away | .35 (<.0001) | .04 (0.5295) | .10 (0.1205) | .29 (<.0001) | .09 (0.1755) | .15 (0.0203) | .14 (0.0306) |
| **Abdominal Scale** | .15 (0.0224) | .54 (<0.0001) | .02 (0.7327) | .48 (<0.0001) | .27 (<0.0001) | .26 (<0.0001) | .24 (0.0003) |
| Passing gas | .17 (0.0116) | .26 (<.0001) | .06 (0.3860) | .30 (<.0001) | .10 (0.1423) | .20 (0.0020) | .13 (0.0504) |
| Pain in belly | .14 (0.0306) | .25 (0.0002) | .06 (0.3620) | .38 (<.0001) | .30 (<.0001) | .15 (0.0263) | .23 (0.0005) |
| Bloating | .07 (0.2962) | .69 (<.0001) | .01 (0.9363) | .43 (<.0001) | .26 (<.0001) | .27 (<.0001) | .20 (0.0020) |
| **Systemic Symptoms** | .21(0.0018) | .34 (<0.0001) | .04 (0.5856) | .40 (<0.0001) | .51 (<0.0001) | .38 (<0.0001) | .36 (<0.0001) |
| Pain in knees, hips, and/or elbows | .07 (0.2692) | .24 (0.0003) | .10 (0.1170) | .30 (<.0001) | .27 (<.0001) | .28 (<.0001) | .17 (0.0107) |
| Feel tired | .23 (0.0005) | .26 (<.0001) | .02 (0.7520) | .29 (<.0001) | .45 (<.0001) | .29 (<.0001) | .26 (<.0001) |
| Feel weak | .17 (0.0091) | .27 (<.0001) | .02 (0.7774) | .30 (<.0001) | .40 (<.0001) | .34 (<.0001) | .38 (<.0001) |
| Lack appetite | .15 (0.0266) | .21 (0.0016) | .00 (0.9696) | .30 (<.0001) | .40 (<.0001) | .24 (0.0002) | .31 (<.0001) |
| Feel thirsty | .16 (0.0138) | .22 (0.0010) | .06 (0.4031) | .24 (0.0003) | .26 (<.0001) | .23 (0.0006) | .15 (0.0196) |

^1^ Spearman's correlation coefficient

Abbreviations: CD-PRO/SS = Crohn’s disease patient-reported outcome signs and symptoms; IBDQ = Inflammatory Bowel Disease Questionnaire; p = p-value; r = rating

Table S9. Construct Validity: CD-PRO/SS and Systemic Symptoms Score Correlations^1^ with EQ-5D-3L Scores at Baseline

| **Domain** | **EQ-5D-3L r (p)** | |
| --- | --- | --- |
|  | **VAS** | **Index** |
| **Bowel Signs and Symptoms** | .15 (0.0260) | -.02 (0.7953) |
| Number of bowel movements | .07 (0.3076) | .12 (0.0808) |
| Mostly liquid bowel movements | .15 (0.0262) | .01 (0.8703) |
| Need to have bowel movement right away | .11 (0.0918) | .10 (0.1155) |
| **Abdominal Scale** | .12 (0.0791) | .32 (<0.0001) |
| Passing gas | .03 (0.6287) | .09 (0.1555) |
| Pain in belly | .22 (0.0008) | .37 (<.0001) |
| Bloating | .10 (0.1445) | .30 (<.0001) |
| **Systemic Symptoms** | .35 (<0.0001) | .54 (<0.0001) |
| Pain in knees, hips, and/or elbows | .20 (0.0027) | .40 (<.0001) |
| Feel tired | .30 (<.0001) | .38 (<.0001) |
| Feel weak | .33 (<.0001) | .46 (<.0001) |
| Lack appetite | .30 (<.0001) | .42 (<.0001) |
| Feel thirsty | .10 (0.1252) | .26 (<.0001) |

^1^ Spearman's correlation coefficients

Abbreviations: CD-PRO/SS = Crohn’s disease patient-reported outcome signs and symptoms; EQ-5D-3L = EuroQol Five Dimensions three levels questionnaire; p = p-value; r = rating; VAS = visual analog scale

Table S10. Known-Groups Validity: CD-PRO/SS and Systemic Symptoms Scores at Baseline by Patient Global Rating of Disease Severity at Baseline

| **Domain Score** | **Patient Global Rating of Disease Severity Score** | | | |  | | |
| --- | --- | --- | --- | --- | --- | --- | --- |
|  | **Very Mild/Mild n, Mean (SD)** | **Moderate n, Mean (SD)** | **Severe n, Mean (SD)** | **Very Severe n, Mean (SD)** | **Overall F value (P value)^1^** | **Pairwise Comparison^2^** | **Cohen's Effect Size** |
| Bowel Signs and Symptoms | 7, 2.2 (0.90) | 88, 2.4 (0.97) | 114, 2.9 (0.88) | 20, 2.8 (1.12) | 0.0054 | 1: 0.960 2: 0.377 3: 0.598 4: 0.013 5: 0.484 6: 0.989 | 1:0.21 2:0.73 3:0.53 4:0.48 5:0.37 6:-0.09 |
| Abdominal Scale | 7, 1.5 (1.05) | 88, 1.7 (0.78) | 114, 2.1 (0.77) | 20, 2.0 (0.84) | 0.0078 | 1: 0.867 2: 0.264 3: 0.583 4: 0.022 5: 0.737 6: 0.926 | 1:0.33 2:0.78 3:0.54 4:0.45 5:0.28 6:-0.17 |
| Systemic Symptoms | 7, 0.9 (0.82) | 88, 1.2 (0.71) | 114, 1.6 (0.77) | 20, 1.7 (0.76) | <0.0001 | 1: 0.874 2: 0.125 3: 0.098 4: 0.001 5: 0.021 6: 0.921 | 1:0.34 2:0.91 3:1.07 4:0.61 5:0.81 6:0.16 |

^1^ Analysis of variance (ANOVA) model.

^2^ 1=Group 1 vs. Group 2, 2=Group 1 vs. Group 3, 3=Group 1 vs. Group 4, 4=Group 2 vs. Group 3, 5=Group 2 vs. Group 4, 6=Group 3 vs. Group 4.

Abbreviations: CD-PRO/SS = Crohn’s disease patient-reported outcome signs and symptoms; SD = standard deviation

Figure S3. Final Conceptual Framework for the CD-PRO/SS

Number of bowel movements

Frequency of bowel movements mostly or completely liquid

Severity of need to have bowel movements right away

Bowel Signs and Symptoms Scale

Frequency of passing gas

Severity of pain in belly

Severity of bloating in belly

Abdominal Symptoms Scale

**General Concepts**

**Item-Level Concept**

Figure S4. Final Conceptual Framework for the Systemic Symptoms Scale

Severity of pain in knees, hips, and/or elbows

Severity of feeling tired

Severity of lack of appetite

Severity of feeling weak

Severity of feeling thirsty

Systemic Symptoms Scale

**General Concepts**

**Item-Level Concept**
